# Evaluating the dual-energy iterative reconstruction algorithm (DIRA) for accurate CT number determination in DECT imaging

**DOI:** 10.1093/rpd/ncaf140

**Published:** 2026-03-13

**Authors:** Maria Magnusson, Michael Sandborg, Åsa Carlsson Tedgren, Alexandr Malusek

**Affiliations:** Department of Electrical Engineering, Linköping University, SE-581 83 Linköping, Sweden; Center for Medical Image Science and Visualization (CMIV), Linköping University, SE-581 85 Linköping, Sweden; Center for Medical Image Science and Visualization (CMIV), Linköping University, SE-581 85 Linköping, Sweden; Department of Health, Medicine and Caring Sciences, Linköping University, SE-581 83 Linköping, Sweden; Department of Medical Physics, Linköping University Hospital, SE-581 85 Linköping, Sweden; Center for Medical Image Science and Visualization (CMIV), Linköping University, SE-581 85 Linköping, Sweden; Department of Health, Medicine and Caring Sciences, Linköping University, SE-581 83 Linköping, Sweden; Department of Nuclear Medicine and Medical Physics, Karolinska University Hospital, SE-171 77 Stockholm, Sweden; Center for Medical Image Science and Visualization (CMIV), Linköping University, SE-581 85 Linköping, Sweden; Department of Health, Medicine and Caring Sciences, Linköping University, SE-581 83 Linköping, Sweden

## Abstract

Accurate computed tomography (CT) numbers derived from dual-energy CT (DECT) have numerous applications, including optimising the precision of radiation treatment planning. The dual-energy iterative reconstruction algorithm (DIRA) utilizes material decomposition in the iterative loop to generate monoenergetic images free of beam-hardening artifacts. In simulations, the reconstructed CT numbers closely matched reference tabulated values. This study evaluates the feasibility of applying DIRA to experimental data. Transitioning from simulations required several adaptations: (i) removal of the patient table, (ii) modeling of the bow-tie filter, and (iii) application of an inverse water beam-hardening correction. Axial scans of a cylindrical polymethyl methacrylate phantom containing four rod inserts of distinct materials were acquired using a clinical Siemens SOMATOM Force scanner. By the sixth iteration, DIRA yielded CT numbers that closely matched tabulated values. The algorithm also demonstrated strong performance on an anthropomorphic head phantom (Computerized Imaging Reference Systems, Inc. (CIRS) model 731-HN) with known material composition.

## Introduction

Computed tomography (CT) reconstructs the linear attenuation coefficients (LACs) of materials, which characterize their X-ray absorption properties. However, because LACs strongly depend on photon energy, projection data are typically corrected using a water beam-hardening correction to suppress associated artifacts. This correction ensures that a phantom composed entirely of water exhibits no beam-hardening artifacts in the reconstructed images. The reconstructed LACs are presented to the end user as CT numbers, defined as:


1 \begin{eqnarray*}& H = 1000 \cdot \left( \frac{\mu}{\mu_{w}} - 1 \right),\end{eqnarray*}

where $\mu$ and $\mu _{w}$ denote the LACs of the material and water, respectively, at a specific photon energy. As a result, the CT number of water is standardized to 0 Hounsfield units (HU).

Clinical X-ray beams are polychromatic, and LACs $\mu (E)$ are energy-dependent. In single energy CT, polychromy causes beam-hardening, where lower-energy photons are preferentially absorbed, producing cupping and streaks when heterogeneous materials are present. A conventional water beam-hardening correction (WBHC) linearizes water-equivalent thickness, but cannot fully correct objects composed of multiple dissimilar materials. Dual energy CT (DECT) acquires two spectra, which allows for better estimation of material-dependent attenuation and can therefore mitigate beam-hardening and improve quantitative CT numbers. One promising application is in radiation treatment planning, where DECT can improve and optimize dosimetric accuracy [1].

DECT algorithms are generally classified into projection-based basis material decomposition (PBBMD) and image-based basis material decomposition (IBBMD) approaches [2]. PBBMD techniques, such as the widely used Alvarez–Macovski base material decomposition (AMBMD) [3], require geometrically consistent projection data and are limited to representing the entire object with only two base materials. In contrast, IBBMD methods allow for more flexibility by incorporating tissue-specific material doublets or triplets. However, they remain susceptible to beam-hardening artifacts due to their image-domain processing.

In [4], two monoenergetic images are generated using the AMBMD method with a water–iodine basis pair. A subsequent three-material decomposition is applied, where pixel-specific material bases are selected based on the reconstructed LACs.

A clinical implementation using IBBMD and the water–iodine basis pair is the Monoenergetic Plus algorithm [5], which we evaluated in [6]. The algorithm performed well overall; however, minor discrepancies in CT numbers were observed, likely due to limitations inherent in the two-material iodine–water decomposition. Iodine presents additional challenges due to its K-edge at 33.2 keV [7, 8]. Furthermore, residual beam-hardening artifacts remained in the reconstructed images, indicating incomplete artifact suppression. This was also observed in [9].

Two methods that simultaneously operate on both projection and image data are material decomposition from inconsistent rays (MDIR) [10] and dual-energy iterative reconstruction algorithm (DIRA) [11]. Both are capable of processing inconsistent rays produced by dual-source DECT scanners. MDIR is an iterative image reconstruction algorithm that generates two base material images using filtered backprojection (FBP) and the original measured projections within its iterative loop. The original 2D DIRA algorithm is also iterative and incorporates FBP within each iteration. It applies two- and three-material decomposition schemes to represent body tissues using material doublets or triplets. The 3D version, DIRA-3D, has been extended to accommodate dual-energy, dual-source helical CT data [12]. Simulation studies have demonstrated that both DIRA and DIRA-3D are capable of producing accurate CT numbers.

This study aims to evaluate the performance of DIRA on real-world DECT data by comparing the reconstructed CT numbers with reference values obtained from the EPDL97 library [13].

Accurate CT numbers from DECT support quantitative applications such as proton range prediction and tissue characterization [1]. The significance of this study lies in its support for DIRA as a practical approach to achieving more quantitative DECT in routine environments.

## Theory

### Material decomposition

Two-material decomposition (2MD) assumes that each voxel consists of a mixture of two base materials. This method estimates their respective mass fractions, $w_{1}$ and $w_{2}$, as well as the total mass density $\rho$ of the mixture. In contrast, three-material decomposition (3MD) models each voxel as a combination of three materials, determining the mass fractions $w_{1}$, $w_{2}$, and $w_{3}$. The corresponding mass density is computed using the relation: $\rho ^{-1} = \sum _{k = 1}^{3} w_{k} / \rho _{k}$, where $w_{k}$ and $\rho _{k}$ are the mass fraction and mass density, respectively, of the $k$th material. In both approaches, the mass fractions are constrained by the normalization condition $\sum _{k} w_{k} = 1$. For a detailed formulation of the underlying system of linear equations, refer to [11].

In the current implementation, decomposition schemes are adapted to tissue type: bone tissues are decomposed into bone, bone marrow, and density; soft tissues into lipid, protein, and water; and low-attenuation materials such as air into lipid, water, and density. However, for the two phantoms examined in this study, such complexity is not required. As demonstrated in [7] and [8], 2MD using a water–bone basis is sufficient to model human tissues with atomic number $Z\leq 20$, covering the materials present in both phantoms.

### Forward projection generation

The logarithm of attenuation, here referred to as the polyenergetic projection $P$, is calculated as


2 \begin{eqnarray*}& P = \ln \frac{I_{0}}{I},\end{eqnarray*}

where $I$ and $I_{0}$ are the detector responses with and without the imaged object. The intensity $I_{0}$, is calculated for an energy-integrating detector as


3 \begin{eqnarray*}& I_{0} = \int_{0}^{E_{max}} D(E) N(E) dE,\end{eqnarray*}

where $D(E)$ is the detector responsivity, $N(E) \, dE$ is the number of photons with energies in the interval ($E$, $E+\, dE$) emitted from the X-ray tube. An ideal detector has $D(E)=E$. The intensity $I$ is calculated as


4 \begin{eqnarray*}& I = \int_{0}^{E_{max}} D(E) N(E) \exp \left[- \int_{\mathcal{L}} \mu (x,y,E) dl\right] dE,\end{eqnarray*}

where $\mu (x,y,E)$ is the LAC of the pixel at position $(x,y)$ with energy $E$ and $\int _{\mathcal{L}} dl$ is a line integral through the object. This calculation is time-consuming since the line integrals must be calculated for all energies in the energy spectrum. The calculation of projections changes slightly when material decomposition is introduced. The line integrals are calculated through volume fractions of the different base materials. The intensity $I$ is then


5 \begin{eqnarray*}& I = \int_{0}^{E_{max}} D(E) N(E) \exp \left[- \sum_{k} \mu_{k}(E)l_{k}\right] dE,\end{eqnarray*}

where $\mu _{k}$ is the LAC of the $k$th base material and $l_{k}$ is computed as


6 \begin{eqnarray*}& l_{k} = \rho^{-1}_{k} \int_{\mathcal{L}} \rho(x,y)w_{k}(x,y) dl,\end{eqnarray*}

where $\rho _{k}$ is the tabulated density of the $k$th base material, $\rho (x,y)$ is the calculated density in the pixel at position $(x,y)$ and $w_{k}(x,y)$ is the mass fraction of the $k$th material in the pixel at position $(x,y)$. The density $\rho$ and the mass fractions $w_{k}$ are obtained from the 2MD or 3MD. The line integral $\int _{\mathcal{L}} dl$ can be calculated using Joseph’s method [14].

A monoenergetic projection $P_{mono}$, at a specific energy $E$, is calculated as


7 \begin{eqnarray*}& P_{mono} = \sum_{k} \mu_{k}(E) l_{k}.\end{eqnarray*}

### Calculation of the two effective energies to be used in DIRA

Two effective energies $E_{\mathrm{eff,L}}$ and $E_{\mathrm{eff,H}}$, where $L$ stands for low and $H$ stands for high, are chosen in DIRA by using the following method. The effective attenuation coefficient $\mu _{\mathrm{eff}}$ for water is calculated by using the weighted mean of the X-ray spectrum and the attenuation curve $\mu (E)$ for water,


8 \begin{eqnarray*}& \mu_{\mathrm{eff}} = \frac{\int_{0}^{E_{max}}D(E)\,N(E)\;\mu(E)\;dE} {\int_{0}^{E_{max}}D(E)\,N(E)\;dE}.\end{eqnarray*}

Then, using the $\mu (E)$-curve for water, the energy value corresponding to $\mu _{\mathrm{eff}}$ is taken as the effective energy. The effective energies $E_{\mathrm{eff,L}}$ and $E_{\mathrm{eff,H}}$ are obtained by setting $N(E)=N_{L}(E)$ and $N(E)=N_{H}(E)$, respectively, in (8).

### Summary of DIRA

**Figure 1:**
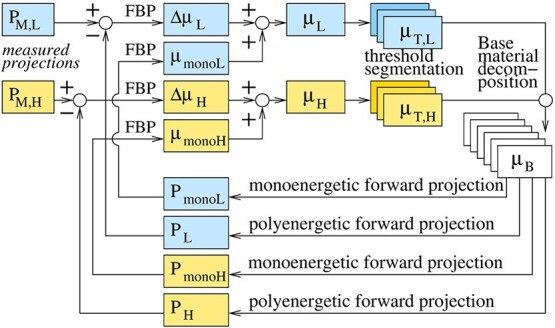
Flowchart of the dual-energy iterative reconstruction algorithm DIRA.

The complete DIRA algorithm is illustrated in Fig. 1 and described below.

1. Measured projections $P_{\mathrm{M,L}}$ and $P_{\mathrm{M,H}}$ are obtained for low- and high-energy spectra, respectively.
2. They are reconstructed with FBP to corresponding $\mu _{\mathrm{L}}$ and $\mu _{\mathrm{H}}$. The initial reconstruction (iteration 0) is preceded by a conventional water beam-hardening correction (not shown in the figure).
3. For the phantoms considered in this work, a threshold segmentation is performed to separate regions of bone tissue, soft tissue, and low attenuation materials such as air. The results are stored in $2\times 3$ images $\mu _{\mathrm{T,L}}$ and $\mu _{\mathrm{T,H}}$.
4. Tissues are decomposed using material decomposition methods, 2MD and 3MD. Density and base material mass fraction images $\mu _{B}$ are received.
5. Monoenergetic forward projections, $P_{\mathrm{mono,L}}$ and $P_{\mathrm{mono,H}}$, are generated for the low and high effective energies, $E_{\mathrm{eff,L}}$ and $E_{\mathrm{eff,H}}$, respectively, see (7) and (8). They are then reconstructed to $\mu _{\mathrm{mono,L}}$ and $\mu _{\mathrm{mono,H}}$.
6. Polyenergetic forward projections $P_{\mathrm{L}}$ and $P_{\mathrm{H}}$ are generated, see (2), (3), and (5). They are then compared with the measured projections. Reconstruction by FBP gives updates $\Delta \mu _{\mathrm{L}}$ and $\Delta \mu _{\mathrm{H}}$. The addition to $\mu _{\mathrm{mono,L}}$ and $\mu _{\mathrm{mono,H}}$ gives $\mu _{\mathrm{L}}$ and $\mu _{\mathrm{H}}$ for the next iteration.

Points 2–6 are iterated a predefined number of times.

## Materials and methods

### CT Scanner and phantom data

**Figure 2:**
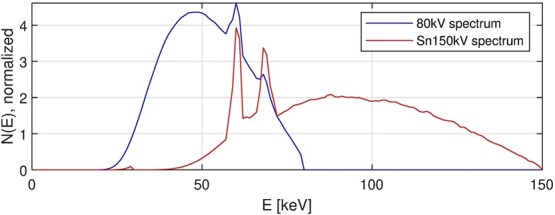
Normalized energy distributions of photons in the two X-ray spectra.

A Siemens SOMATOM Force dual-source CT scanner (Siemens Healthineers) was used to acquire axial dual-energy scans of a cylindrical polymethyl methacrylate (PMMA) phantom ($\phi$160 mm) containing four cylindrical rod inserts ($\phi$20 mm): one aluminum, two polytetrafluoroethylene (PTFE, Teflon), and one low-density polyethylene (LDPE), see Fig. 4, top left. Four rods were used to make the phantom more interesting and to induce beam-hardening artifacts between the aluminum and PTFE rods. Peripheral cavities designed for pencil ion chambers were filled with air. A clinical-style, axial dual-energy protocol was employed using tube voltages of 80 and 150 kV with an added tin (Sn) filter (denoted Sn150 kV), see Fig. 2, The corresponding effective photon energies were 50 and 93 keV, respectively, see (8).

For comparison, images were reconstructed using the Advanced Modeled Iterative Reconstruction (ADMIRE) algorithm (Qr36d kernel, strength 2) within Siemens’ syngo.via version 5.1 software. ADMIRE substantially reduces image noise compared to conventional FBP. In our reconstructions with DIRA, applied to the rod phantom, several detector rows were averaged to further reduce noise, an appropriate approach given the phantom’s uniformity along the longitudinal axis.

**Figure 3:**
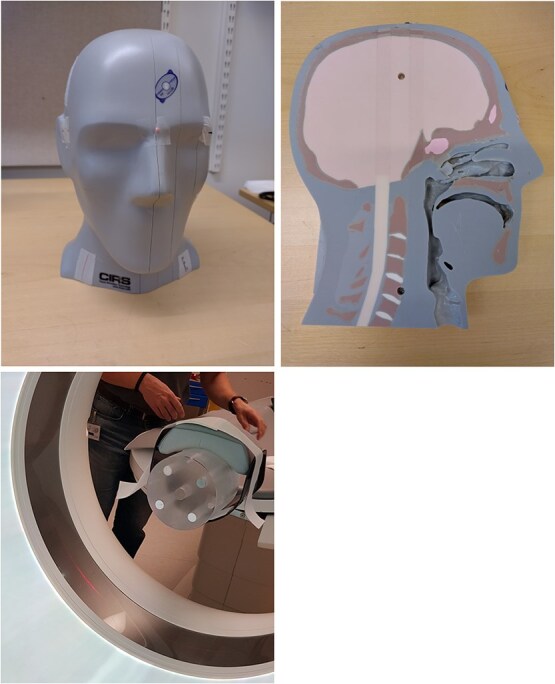
Top: The CIRS Model 731-HN head phantom. Bottom: The rod phantom floating in the center of the CT scanner.

Additionally, an anthropomorphic head phantom (CIRS model 731-HN), shown in Fig. 3, was scanned axially at the level of the teeth. The CIRS head phantom has a specified material composition corresponding to brain, soft tissue, spinal cord, trabecular bone, cortical bone, enamel, dentin, and more.

**Figure 4:**
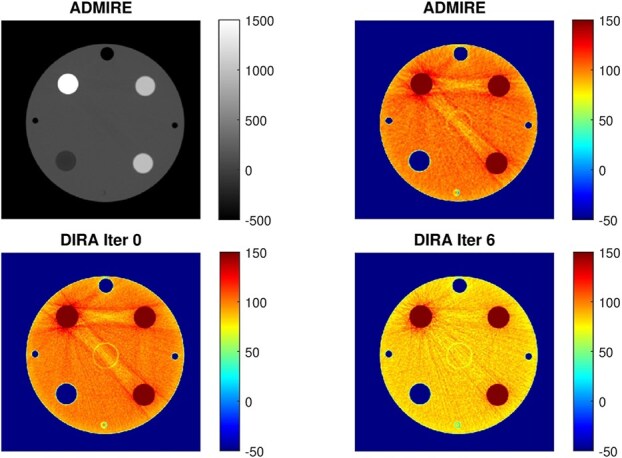
Rod phantom scanned at 80 kV, 50 keV effective energy, and reconstructed by ADMIRE and DIRA at iterations 0 and 6. The phantom consists of a PMMA cylinder with four rod inserts: Al (top-left), LDPE (bottom-left), and two PTFE rods (right). Three smaller cavities are filled with air.

### DIRA adaptations for experimental data

See Fig. 1. To obtain input data consisting of the measured projections $P_{\mathrm{M,L}}$ and $P_{\mathrm{M,H}}$, the following procedure was performed. The CT scanner was configured for axial acquisition, and raw projection data were exported. These data may be treated as fanbeam projections. The fanbeam projections were subsequently rebinned into parallel-beam geometry using 511 detector channels per projection angle. These constitute the measured projections $P_{\mathrm{M,L}}$ and $P_{\mathrm{M,H}}$ at low and high energies, respectively.

Unfortunately, the rawdata had already undergone water beam correction (WBHC), which was not suitable for DIRA, as the algorithm is designed to work on uncorrected data. Previous attempts to disable WBHC via service mode failed. Therefore, an inverse WBHC procedure was designed and applied to restore the original, uncorrected projections. We derived a monotonic 1D look-up function that maps corrected line integrals back to water-equivalent pathlengths before correction. The function can be easily calculated using the current polyenergetic spectrum and energy-dependent LAC data for water. The two different basic spectra (low- and high-energy) further change when travelling through the bowtie filter and depending on the angle, see below. The spectra and bowtie filter specification were obtained from Siemens, and the water data were obtained from the EPDL97 library [13].

To eliminate the influence of the patient table, two approaches were tested with the rod phantom: (i) mounting the phantom at the end of the table to allow it to ‘float’ in the scanner’s isocenter (see Fig. 3) and (ii) acquiring a reference scan of the table alone and subtracting it from a scan with the phantom on the table. Both approaches yielded comparable results.

Special care was taken to account for angular variation in the X-ray spectrum. The bowtie filter is made of aluminum and is thinnest along the central axis. As the X-rays travel through the bowtie filter, they are attenuated and undergo beam-hardening. Therefore, the shape of the spectrum varies depending on the angle at which the rays are emitted from the X-ray tube. As a result, the effective spectrum D(E)N(E) differs with projection angle, see (3)–(5). DIRA was adapted to model and incorporate this angular dependence into its reconstruction process.

For the CIRS head phantom, the scan was performed with the phantom placed on a pillow on top of the patient table. A follow-up scan of the table and pillow alone was subtracted from the initial dataset to isolate the phantom. However, slight differences in the pillow’s position and shape between scans introduced a subtle artifact in the posterior head region.

**Figure 5:**
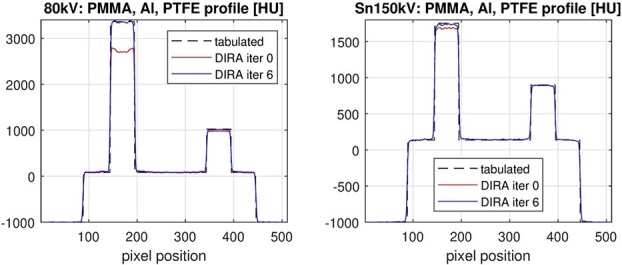
Profiles along the Al and PTFE rods in the PMMA rod phantom for the two spectra, 80 kV and Sn150 kV.

**Table 1:** CT numbers [HU] calculated in regions of radius 10 pixels in the different materials in the rod phantom, along with tabulated values. 80 kV spectrum with effective energy 50 keV. Values are mean $\pm$ standard deviation over all pixels in the stated region.

Material	ADMIRE	DIRA, iter = 0	DIRA, iter = 6	Tabulated
Al	2711	2700 $\pm$ 8	3341 $\pm$ 11	3363
PTFE	983	980 $\pm$ 3	1010 $\pm$ 5	1026
PMMA	104	102 $\pm$ 3	85 $\pm$ 4	83
LDPE	–111	–114 $\pm$ 2	–134 $\pm$ 3	–135

## Results and discussion

Figure 4 and Table 1 present results for the rod phantom reconstructed using ADMIRE and DIRA at iterations 0 and 6, for the 80 kV spectrum with an effective energy of 50 keV. Table 1 shows that DIRA at iteration 6 yielded CT numbers close to tabulated reference values. Moreover, ADMIRE and DIRA at iteration 0 produced close numerical agreement and visually similar results, indicating consistency between our FBP implementation and the commercial reconstruction. Also note that at iteration 6, DIRA succeeded in suppressing the beam-hardening artifacts between the aluminum and PTFE rods. It is noteworthy that this artifact remained partially unresolved using Monoenergetic Plus in prior work [6].

**Figure 6:**
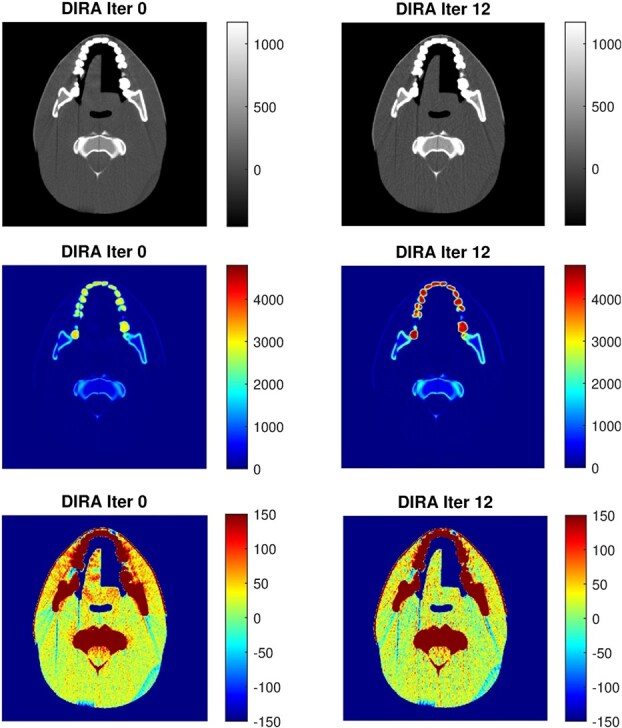
Images of the CIRS head phantom reconstructed by DIRA at iterations 0 and 12. 80 kV spectrum with effective energy 50 keV. Different contrast windows in grayscale and color are used to visualize the result: Iteration 12 improves (increases) CT values in bone/teeth, suppresses beam-hardening artifacts, and improves soft-tissue homogeneity (compare tongue and neck).

Figure 5 displays line profiles across the aluminum and PTFE rods in the PMMA phantom, acquired at 80 kV (effective energy 50 keV) and Sn150 kV (effective energy 93 keV). For the aluminum rod, a clear progression toward the tabulated attenuation values is observed with increasing DIRA iterations. In particular, at 80 kV, the downward curvature apparent in iteration 0 is effectively corrected by iteration 6, yielding a more uniform and accurate profile. This illustrates DIRA’s capability to reduce beam-hardening artifacts and enhance quantitative accuracy.

Figure 6 displays axial images of the CIRS head phantom reconstructed using DIRA at iterations 0 and 12, based on the 80 kV spectrum with an effective energy of 50 keV. The images are shown using three different contrast windows and corresponding color tables. In the wide contrast window (top row), minimal visual differences are observed between iterations. However, when the contrast is optimized for bone and teeth (middle row), and the color table adjusted accordingly, a clear increase in CT values is apparent with increasing iteration number. Under a narrow contrast window (bottom row), both image noise and beam-hardening artifacts become more prominent. Notably, iteration 6 yielded a more uniform soft tissue tone, particularly in the halved tongue region, indicating improved homogeneity.

Table 2 quantifies the improvement in CT number accuracy from iteration 0 through iterations 6 and 12, in comparison with values from the CIRS phantom specification. It is clear that (i) the enamel error decreased markedly with iteration (iter 0 $\Rightarrow$ 6/12), and (ii) the two soft tissue ROIs (tongue, neck) converged to almost identical CT numbers (with a difference of only 1 HU at iter 12), although a common soft tissue bias of approximately +10 HU remained relative to the specification. It should be mentioned that we do not know how precise the specification for the CIRS head phantom is. The corresponding values for the rod phantom are much more reliable because we fabricated it ourselves from very pure materials.

**Table 2:** CT numbers [HU] calculated with DIRA in circular regions of radius 10 pixels (6 for enamel) in the CIRS phantom, along with specified values. 80 kV spectrum with effective energy 50 keV. Values are mean $\pm$ standard deviation over all pixels in the stated region.

Tissue	DIRA, iter = 0	DIRA, iter = 6	DIRA, iter = 12	Specified
Trabecular bone	426 $\pm$ 25	526 $\pm$ 36	526 $\pm$ 36	488
Enamel	3183 $\pm$ 42	4560 $\pm$ 64	4595 $\pm$ 65	4720
Tongue	78 $\pm$ 24	34 $\pm$ 24	30 $\pm$ 23	19
Neck	28 $\pm$19	30 $\pm$27	29 $\pm$27	19

## Conclusion

The DIRA demonstrated strong performance on experimentally acquired dual-energy CT data. The reconstructed CT numbers showed close agreement with tabulated reference values from the EPDL97 database, confirming DIRA’s quantitative accuracy. Image-based evaluations further indicated effective suppression of beam-hardening artifacts with increasing iterations.

While DIRA achieved noise levels comparable to those of FBP, it did not match the noise reduction performance of ADMIRE, which incorporates advanced denoizing techniques. Nonetheless, DIRA provides a promising framework for accurate CT number reconstruction in dual-energy imaging, particularly in scenarios where artifact correction is prioritized.

Future work will (i) include noise reduction inspired by [15] and Siemens’ ADMIRE, (ii) extend evaluations to helical acquisitions, and (iii) extend evaluations to iodine-containing tasks.
